# Oral ciprofloxacin biofilm activity in a catheter-associated urinary tract infection model

**DOI:** 10.1093/jac/dkae424

**Published:** 2024-11-29

**Authors:** Iain J Abbott, Connor R B Anderson, Elke van Gorp, Steve C Wallis, Jason A Roberts, Joseph Meletiadis, Anton Y Peleg

**Affiliations:** Department of Infectious Diseases, Alfred Hospital and School of Translational Medicine, Monash University, Melbourne, Victoria, Australia; Department of Infectious Diseases, Alfred Hospital and School of Translational Medicine, Monash University, Melbourne, Victoria, Australia; Department of Infectious Diseases, Alfred Hospital and School of Translational Medicine, Monash University, Melbourne, Victoria, Australia; University of Queensland Centre for Clinical Research, Faculty of Medicine, The University of Queensland, Brisbane, Australia; University of Queensland Centre for Clinical Research, Faculty of Medicine, The University of Queensland, Brisbane, Australia; Clinical Microbiology Laboratory, Attikon University Hospital, Medical School, National and Kapodistrian University of Athens, Haidari, Athens, Greece; Department of Infectious Diseases, Alfred Hospital and School of Translational Medicine, Monash University, Melbourne, Victoria, Australia; Department of Microbiology, Infection Program, Monash Biomedicine Discovery Institute, Monash University, Clayton, Victoria, Australia

## Abstract

**Background:**

Catheter-associated urinary tract infections (CA-UTIs) are a common hospital-acquired infection. We examined ciprofloxacin activity in a novel CA-UTI *in vitro* model.

**Methods:**

Three ATCC strains [*Escherichia coli* (ECO)-25922, *Klebsiella pneumoniae* (KPN)-700721, *Pseudomonas aeruginosa* (PAE)-27853] and 45 clinical urinary isolates were assessed. Biofilm mass and planktonic bacterial density were quantified during drug-free incubation (72 h) and following ciprofloxacin exposure (equivalent 750 mg orally q12h, 3 days).

**Results:**

ECO produced smaller biofilms (6.3 ± 1.1 log_10_ cfu/cm^2^) compared with KPN (7.1 ± 0.7 log_10_ cfu/cm^2^) and PAE (7.0 ± 1.2 log_10_ cfu/cm^2^), which extended along the entire catheter length. Following ciprofloxacin, all isolates with MIC > 4 mg/L had minimal biofilm disruption or planktonic kill. Ciprofloxacin resistance was most common in PAE isolates (10/16 isolates), compared with ECO (3/16 isolates) and KPN (6/16 isolates). Greater ciprofloxacin exposure (AUC_0–24_/MIC) was required for a 3 log_10_ biofilm kill for KPN (5858; *R*^2^ = 0.7774) compared with ECO (2117; *R*^2^ = 0.7907) and PAE (2485; *R*^2^ = 0.8260). Due to persistent growth in the bladder, ECO required greater ciprofloxacin exposure for a 3 log_10_ planktonic kill (5920; *R*^2^ = 0.8440) compared with KPN (2825; *R*^2^ = 0.9121) and PAE (1760; *R*^2^ = 0.8781). Monte Carlo simulation supported a 95% PTA for both a 3 log_10_ biofilm and planktonic kill for ECO and KPN isolates with MIC ≤ 0.5 mg/L and PAE isolates with MIC ≤ 1 mg/L.

**Conclusions:**

In a novel CA-UTI model, following simulated ciprofloxacin therapy, KPN biofilms were comparatively more difficult to disrupt, ECO planktonic growth frequently persisted in the bladder, and PAE had greater propensity for emergence of ciprofloxacin resistance.

## Introduction

The optimal antimicrobial therapy for a catheter-associated urinary tract infection (CA-UTI) is uncertain. CA-UTIs account for 26%–40% of all healthcare-associated infections and 75% of hospital-acquired (HA) UTIs.^1–3^ Indwelling urinary catheters are very common in hospitalized patients, with a point prevalence of 19%–26%.^2^ The duration of catheterization is the most important risk factor for CA bacteriuria, with the risk increasing by 3%–8% per day.^3^ HA-UTIs are associated with a range of negative outcomes: increased length of stay in hospital, greater antibiotic usage, patient morbidity and increase in costs.^2,4–7^

A CA-UTI is a UTI in a patient who is catheterized or had a catheter in place within 48 h. Infections involve a broader range of bacterial species compared with uncomplicated infections (uUTIs), are more likely to be polymicrobial and harbour antimicrobial resistance.^8^ Urinary catheter implantation induces a variety of host responses, bladder wound healing and deposition of fibrinogen.^1^ This facilitates bacteria to survive in biofilms—complex microbial communities embedded in an extracellular matrix—that, despite high concentrations of antimicrobials, can lead to treatment failure and infection recurrence.^9,10^

The study of antibiofilm activity of antimicrobials in CA-UTIs is in its infancy.^11^ We demonstrated the use of a dynamic CA-UTI pharmacokinetic/pharmacodynamic (PK/PD) model to simulate uropathogen biofilm formation on urinary catheters and quantify the antimicrobial exposure–response required for both biofilm disruption and planktonic bacterial kill. We assessed oral ciprofloxacin activity against *Escherichia coli* (ECO), *Klebsiella pneumoniae* (KPN) and *Pseudomonas aeruginosa* (PAE) isolates.

## Methods

### Media and antibiotic

The media used were: cation-adjusted Mueller–Hinton II agar (MHA, Becton-Dickinson, USA; used for quantitative cultures), CAMHB (BD; used for MIC susceptibility testing), pooled human urine from healthy female volunteers (ethics #27033; used for drug-free bacterial growth under static incubation conditions in comparison with other media), and modified synthetic human urine (mSHU; pH 5.6),^12^ including casamino acids and yeast extract to match bacterial growth in human urine (see Supplementary methods; available as Supplementary data at *JAC* Online; Table S1 and Figure S1), used for the CA-UTI dynamic model (10 L/day) and for non-standard MIC susceptibility testing.

Two ciprofloxacin formulations were used. Ciprofloxacin HCl (Sigma-Aldrich USA, PHR1044) was used for susceptibility testing. Ciprofloxacin Injection for Intravenous Infusion (Aspen Pharmacare Australia; 200 mg/100 mL) was used to simulate the urinary ciprofloxacin concentrations in the CA-UTI model and in ciprofloxacin-supplemented MHA plates (CIP-MHA-2 mg/L, CIP-MHA-128 mg/L). The IV formulation was incompatible in media with pH ≥6.5 and was used for practical purposes given the large volumes of antibiotic stock required for each experiment.

### Bacterial strains

ATCC strains were used initially (ECO-25922, KPN-700721, PAE-27853). Following initial testing, 45 clinical, non-duplicate isolates from a urinary source (15 each of ECO, KPN and PAE, sourced from a clinical isolate repository; ethics #533/16) were included. The biofilm formation capacity of these isolates was unknown. Susceptibility testing was performed in triplicate by broth microdilution (BMD) in CAMHB using standard methodology (median, range reported).^13^ WT isolates were defined by the epidemiological cut-off (ECOFF) values: ECO 0.06 mg/L, KPN 0.125 mg/L, PAE 0.5 mg/L.^14^ EUCAST susceptibility test interpretive criteria were applied: MIC ≤ 0.5 mg/L considered susceptible, increased exposure.^15^ A MIC value of 1–4 mg/L was considered as low-level resistant (LLR), and a MIC > 4 mg/L as high-level resistant (HLR). MICs were also measured in mSHU.

### CA-UTI model

**Figure 1.**
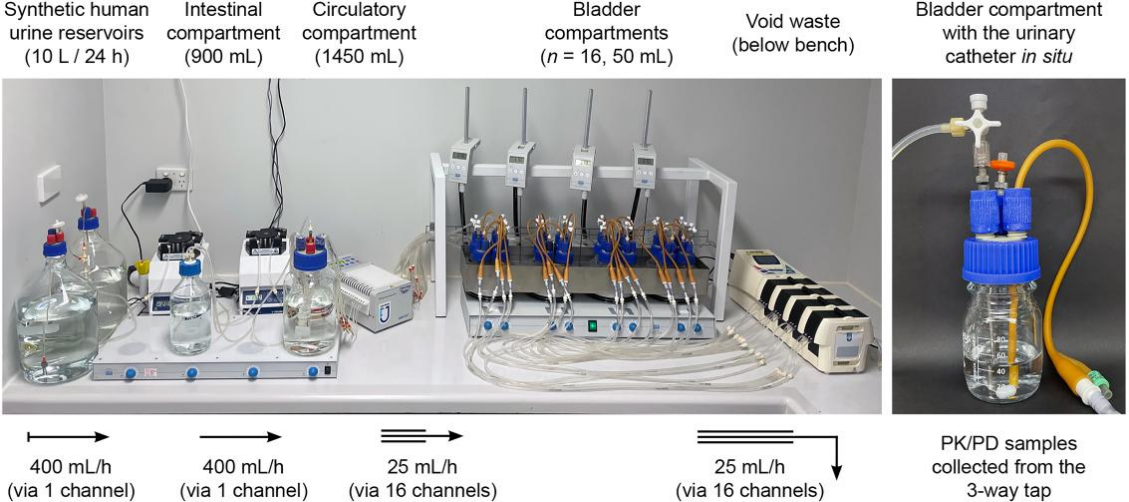
CA-UTI biofilm model. The set-up of the dynamic multicompartment dilutional *in vitro* model used for the simulation of a catheter-associated UTI (CA-UTI) and treatment with ciprofloxacin following oral dosing. The void waste compartment is not shown and is located below the laboratory bench. This figure appears in colour in the online version of *JAC* and in black and white in the print version of *JAC*.

A multicompartment continuous dilution model was adapted from previously.^16^ The set-up (Figure 1) is described in greater detail in the Supplementary methods. In contrast to the original uUTI model, each bladder compartment was prepared with a urinary catheter [latex Foley urinary catheter, 14 Ch/Fr (4.7 mm); Bard^®^, USA] aseptically placed in the outflow position with continuous drainage, bladder volume maintained at 50 mL, and each bladder inoculated with 10 mL of 10^6^ cfu/mL inoculum added to 40 mL of mSHU, providing a total bacterial count equivalent to human UTIs (i.e. ≥10^5^ cfu/mL in 200 mL void). The model was run for 72 h prior to ciprofloxacin administration to allow time for biofilm formation.

### Simulated ciprofloxacin exposure

Dosing was simulated to mimic 750 mg orally 12-hourly for 3 days, with the targeted urinary exposures informed by human data.^17^ This reflects the clinical preference for oral therapy when possible. Greater urinary ciprofloxacin exposure would be expected following IV administration (i.e. 400 mg IV 8-hourly); however, this was not simulated in this study. Protein binding in urine was considered to be minimal. Target peak (*C*_max_) and trough (*C*_min_) concentrations, and the AUC_0–24_ on the third day of treatment were *C*_max_ 645 mg/L, *C*_min_ 95 mg/L, AUC_0–24_ 9000 mg·h/L, and >90% of each dose excreted by 12 h. Ciprofloxacin concentrations were quantified by ultra-high-performance liquid chromatography·with fluorometric detection (UHPLC-Fl). An estimated AUC_0–24_/MIC ratio was calculated for each isolate (see Supplementary methods).

### Bacterial response assessments

Quantitative cultures determined the biofilm mass (cfu/cm^2^, in triplicate) and planktonic bacterial density in the bladder (cfu/mL) during 72 h drug-free incubation followed by 72 h ciprofloxacin exposure. Time to biofilm formation was assessed in the ATCC strains with quantitative cultures after 6, 24, 48 and 72 h drug-free incubation. ATCC strains were also assessed by scanning electron microscopy (SEM) to visualize the biofilms and repeat quantitative cultures 24 h after the completion of ciprofloxacin. All isolates had the total population (TP) and the resistant subpopulation (RSP) quantified by plating on MHA and ciprofloxacin-supplemented MHA, respectively (see Supplementary methods). End-of-treatment (EOT) repeat ciprofloxacin MIC testing was performed on both the planktonic and biofilm regrowth. A significant rise in MIC was defined as a >2-fold log_2_ dilution rise from baseline.

### Exposure–response analysis

The relationship between ciprofloxacin exposure (AUC_0–24_/MIC) and bacterial response was quantified using a non-linear regression variable slope *E*_max_ model, described by the equation: *E* = (*E*_max_ − *E*_min_) × EI*^n^*/(EI*^n^* + EI*^n^*_50_) + *E*_min_, where *E*_max_ reflects maximal growth and *E*_min_ reflects no growth, EI is the exposure index, EI_50_ is the exposure index required to achieve 50% of *E*_max_, and *n* is the slope of the dose–effect relationship (Hill coefficient). The goodness of fit was assessed by visual inspection and *R*^2^. The change in biofilm mass (Δlog_10_ cfu/cm^2^) and change in planktonic bacterial density (Δlog_10_ cfu/mL) were calculated from the growth after 72 h drug-free incubation. Each species was analysed separately. Monte Carlo simulations (MCS), using a ‘normal random number generator’ for 5000 patients (Microsoft^®^ Excel for Mac, v16.87), was used to determine the PTA for both effective biofilm and planktonic kill in relation to baseline ciprofloxacin MIC with ±50% allowance in the expected human urinary ciprofloxacin concentrations. All data were analysed using GraphPad Prism (version 10.3.0 macOS).

## Results

### Baseline ciprofloxacin susceptibility

ATCC strains returned ciprofloxacin MIC values within expected targets. Clinical isolates had ciprofloxacin MIC 0.25–512 mg/L for ECO, 0.016–256 mg/L for KPN, and 0.125–32 mg/L for PAE (Table 1). MIC measurements were 4.1 ± 1.0 2-fold log_2_ dilutions higher when tested in mSHU compared with testing in CAMHB (ECO 3.9 ± 1.2; KPN 4.1 ± 1.0; PAE 4.3 ± 0.9) (Table S2).

**Table 1.** Baseline characteristics, drug-free growth and post-exposure response to ciprofloxacin therapy

Isolate #	Baseline		Drug-free growth (at 72 h)		Post CIP exposure (at 144 h)			
			Biofilm	Planktonic	Biofilm		Planktonic	
	CIP MIC, mg/L, median (range)^a^	Interp.	Bacterial mass, log_10_ cfu/cm^2^ (±SD)	Bacterial density, log_10_ cfu/mL	Bacterial mass, log_10_ cfu/cm^2^ (±SD)	CIP MIC, mg/L (TP/RSP)^b^	Bacterial density, log_10_ cfu/mL	CIP MIC, mg/L (TP/RSP)^b^
***E. coli***	**Range: 0.008–512**		**Average: 6.3 ± 1.1**	**Average: 8.1 ± 0.2**				
25 922^c^	0.008 (0.008)	S	6.1 (0.8)	8.1	—	—/—	—	—/—
057	0.25 (0.25)	S	7.0 (0.7)	8.2	—	—/—	3.1	0.25/—
017	0.5 (0.25–0.5)	I	7.0 (0.5)	8.2	—	—/—	2.7	0.5/—
014	0.5 (0.25–0.5)	I	6.0 (0.9)	8.1	2.4 (0.2)	0.5/—	3.7	0.5/—
015	0.5 (0.5)	I	5.9 (0.4)	8.1	—	—/—	6.2	0.5/—
016	0.5 (0.5)	I	5.6 (0.9)	8.1	—	—/—	3.7	0.5/—
019	1 (1)	R	6.6 (0.2)	8.0	—	—/—	4.1	1/—
114	4 (4–8)	R	7.0 (0.8)	8.1	3.8 (0.2)	8/**64**	3.7	**32**/**32**
132	8 (4–8)	R	3.5 (0.7)	7.9	3.7 (0.1)	**64**/32	7.8	32/**64**
104	8 (8)	R	7.1 (1.1)^e^	8.1	4.2 (1.0)	32/32	8.2	32/**64**
093	16 (16)	R	5.6 (0.7)	7.8	6.0 (1.4)^e^	32/32	8.4	64/32
124	32 (32)	R	7.0 (0.2)	8.4	7.3 (1.1)^e^	32/32	9.5	32/32
096	32 (32)	R	6.7 (0.0)	8.2	5.3 (0.4)	64/64	8.5	64/64
127	64 (64)	R	6.1 (0.3)	7.9	5.4 (0.2)	64/64	9.1	64/256
139	128 (128)	R	7.4 (0.2)	8.0	8.1 (1.2)^e^	128/256	8.8	128/256
087	512 (512)	R	7.0 (0.3)	8.3	5.2 (1.3)^e^	>256/>256	8.9	>256/>256
***K. pneumoniae***	**Range: 0.016–256**		**Average: 7.1 ± 0.7**	**Average: 8.9 ± 0.3**				
014	0.016 (0.016–0.03)	S	6.9 (1.0)	8.8	—	—/—	—	—/—
079	0.016 (0.016)	S	7.6 (0.4)	8.7	2.8 (0.5)	0.016/—	—	—/—
161	0.03 (0.03)	S	6.9 (0.5)	8.7	2.5 (0.3)	0.06/—	2.0	0.06/—
174	0.03 (0.016–0.06)	S	7.1 (0.6)	8.3	2.4 (0.2)	0.06/—	—	—/—
348	0.03 (0.03)	S	7.4 (0.7)	8.9	2.9 (0.6)	0.5/—	—	—/—
319^d^	0.125 (0.06–0.125)	S	5.6 (0.4)	8.8	7.8 (0.4)	**128**/**256**	9.0	**128**/**256**
018	0.5 (0.5)	I	6.4 (0.0)	8.8	—	—/—	2.5	1/—
344	0.5 (0.5)	I	7.0 (0.5)	9.1	—	—/—	4.1	0.5/**128**
223	1 (0.5–1)	R	7.5 (0.1)	8.9	2.6 (0.5)	1/—	4.1	1/**16**
322	2 (2)	R	7.3 (0.7)	8.6	6.7 (1.6)^e^	**16**/**16**	5.1	2/**16**
700 721^c^	2 (1–2)	R	7.6 (0.5)	9.7	5.0 (0.8)	**16**/**16**	3.3	**16**/**16**
334	4 (4)	R	7.2 (1.2)^e^	8.7	3.1 (1.1)^e^	4/8	4.8	16/16
171	8 (8)	R	6.7 (1.0)	9.0	6.7 (0.1)	32/32	9.2	16/**128**
076	64 (32–64)	R	7.4 (0.2)	9.0	6.4 (1.0)	64/64	9.2	64/64
044	128 (128)	R	7.1 (0.3)	9.2	7.4 (0.5)	128/128	9.2	128/128
142	256 (256)	R	7.7 (0.3)	9.0	7.7 (0.6)	256/256	9.3	256/256
***P. aeruginosa***	**Range: 0.125–32**		**Average: 7.0 ± 1.2**	**Average: 8.4 ± 0.6**				
21 994	0.125 (0.125–0.25)	I	7.1 (0.1)	8.1	2.7 (0.7)	0.25/—	2.7	0.5/—
50 210	0.125 (0.125–0.25)	I	7.3 (0.4)	7.7	2.6 (0.6)	0.125/—	2.5	**2**/—
83 412	0.25 (0.125–0.25)	I	6.6 (1.8)^e^	8.3	—	—/—	4.9	1/—
81 458	0.25 (0.125–0.5)	I	7.5 (0.4)	8.2	3.2 (0.7)	0.25/—	2.6	**2**/—
01 643	0.25 (0.125–0.5)	I	5.5 (1.1)^e^	8.1	2.6 (0.2)	0.25/—	3.0	0.25/—
63 519	0.25 (0.25)	I	6.5 (0.4)	8.5	—	—/—	—	—/—
27 853^c^	0.25 (0.25–0.5)	I	8.1 (0.4)	9.2	2.5 (0.3)	0.25/—	2.9	**2**/—
12 030	0.5 (0.5)	I	7.6 (0.2)	8.8	—	—/—	—	—/—
91 643	2 (2–4)	R	7.5 (0.3)	9.0	2.5 (0.2)	4/2	3.1	8/**16**
61 963	4 (4)	R	5.6 (1.7)^e^	7.0	—	—/—	—	—/—
44 425	8 (4–8)	R	7.3 (0.3)	8.9	6.1 (0.9)	32/**64**	7.4	32/32
92 669	8 (8–16)	R	4.7 (2.3)^e^	9.1	6.3 (0.6)	16/16	7.0	**64**/**64**
60 707	16 (16)	R	8.0 (0.3)	8.1	5.7 (0.7)	16/16	7.1	**256**/**128**
60 905	16 (16)	R	7.7 (0.1)	8.3	6.5 (0.6)	**128**/32	7.0	**256**/64
27 501	32 (32)	R	7.8 (0.2)	8.1	6.8 (0.7)	**>256**/**>256**	8.3	**>256**/**>256**
87 386	32 (32–64)	R	7.4 (0.2)	8.4	7.6 (0.8)	**>256**/**>256**	8.7	**256**/**>256**

I, susceptible increased exposure; Interp., susceptibility category interpretation; R, resistant; S, susceptibile standard dosing regimen.

^a^Ciprofloxacin (CIP) MIC measured at baseline (in triplicate, interpretation as per EUCAST breakpoint table v 14.0) and post-ciprofloxacin exposure.

^b^Post-exposure MIC performed if there was end-of-treatment biofilm or planktonic growth. The total population (TP, growth on drug-free MHA) and the resistant subpopulation (RSP, growth on ciprofloxacin-supplemented MHA) were measured as a single MIC measurement. A >2-fold dilution rise in MIC compared with baseline is indicated in bold and underlined.

^c^ATCC strains.

^d^KPN*-*319 was found to have an RSP at baseline (MIC 16 mg/L).

^e^Non-confluent biofilm growth (value underlined) indicated by SD of the mean biofilm mass greater than ±1 log_10_ cfu/cm^2^. —, indicates no growth detected at the limit of detection at EOT (either biofilm or planktonic bacterial growth) and indicates where the lack of growth meant that post-exposure MIC testing was not able to be performed (on the TP or RSP).

### Ciprofloxacin exposure in CA-UTI model

The UHPLC-Fl assay performance met FDA guidance (precision 3.5%–6.9%, accuracy 4.5%–10.5%).^18^ Ciprofloxacin concentrations closely matched target values (bias 3.1 ± 16.6% and 44.8 ± 58.7 mg/L; linear regression slope 1.1, *R*^2^ = 0.9722) with minimal intercompartment and interday variation (Figure S2). Average ciprofloxacin concentrations were *C*_max_ 710.0 ± 39.7 mg/L and *C*_min_ 83.6 ± 16.7 mg/L. The average AUC_0–24_ was 10 279 ± 697 mg/L.h.

### ATCC isolates

**Figure 2.**
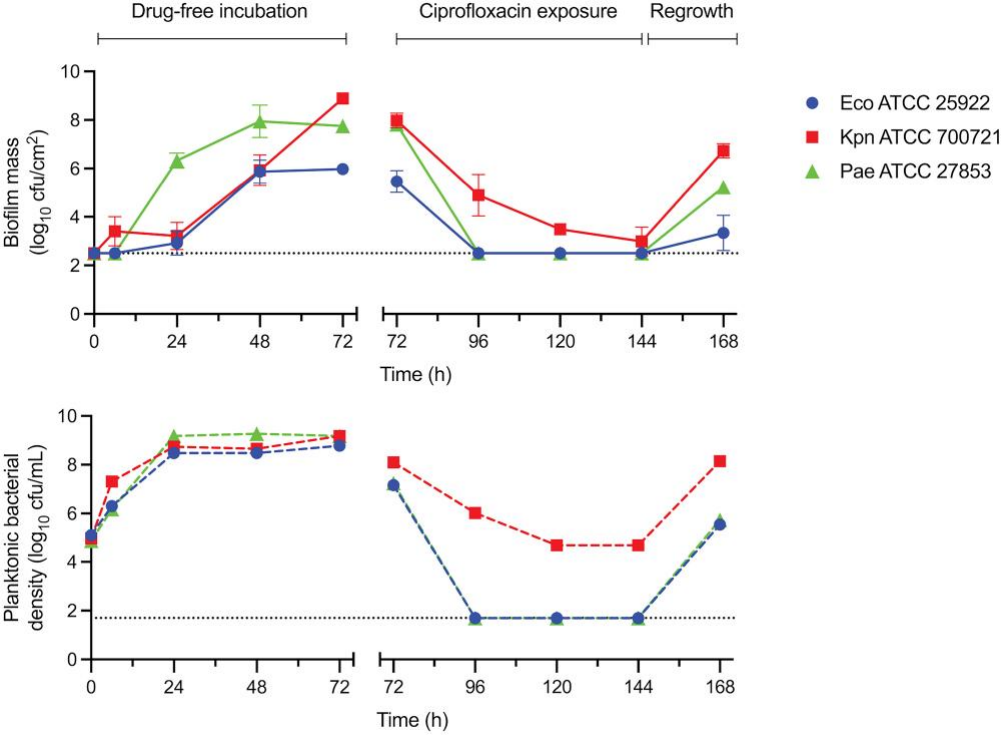
ATCC isolates in CA-UTI model. Drug-free incubation in the model for the initial 72 h, followed by 12-hourly ciprofloxacin administration for 72 h, followed by 24 h incubation without ciprofloxacin administration. Each biofilm mass data point is reflective of a separate bladder-catheter compartment set-up. Error bars represent the standard deviation of the mean biofilm mass quantification tested in triplicate. The drug-free experiment was performed separately from the ciprofloxacin exposure experiment. Scanning electron microscopy images of the dissected catheter (Figure 3) were taken at 72 and 144 h. Dotted line represents the limit of detection of the quantitative cultures. Eco, *Escherichia coli*; Kpn, *Klebsiella pneumoniae*; Pae, *Pseudomonas aeruginosa*. This figure appears in colour in the online version of *JAC* and in black and white in the print version of *JAC*.

After 24 h drug-free incubation under static conditions in 25 mL media, bacterial density in mSHU matched the growth in pooled human urine (Figure S1). The post-incubation pH change in mSHU was most pronounced for KPN-700721 (pH rise to 8.5), compared with PAE-27853 (pH rise to 6.4) and ECO-25922 (pH unchanged at 5.6). After 72 h dynamic drug-free incubation in mSHU in the CA-UTI model, despite all isolates achieving similar planktonic bacterial density (ECO-25922: 8.8 log_10_ cfu/mL; KPN-700721: 9.2 log_10_ cfu/mL, PAE-27853: 9.2 log_10_ cfu/mL; post-incubation pH not measured), biofilm formation differed between the three species (Figure 2). ECO-25922 formed a smaller biofilm (6.0 ± 0.2 log_10_ cfu/cm^2^) compared with KPN-700721 (8.9 ± 0.2 log_10_ cfu/cm^2^) and PAE-27853 (7.8 ± 0.1 log_10_ cfu/cm^2^). After 24 h incubation, ECO-25922 and KPN-700721 had minimal biofilm formation compared with PAE-27853. For KPN-700721, the biofilm mass continued to increase over 72 h incubation, whereas ECO-25922 and PAE-27853 tended to plateau after 48 h.

**Figure 3.**
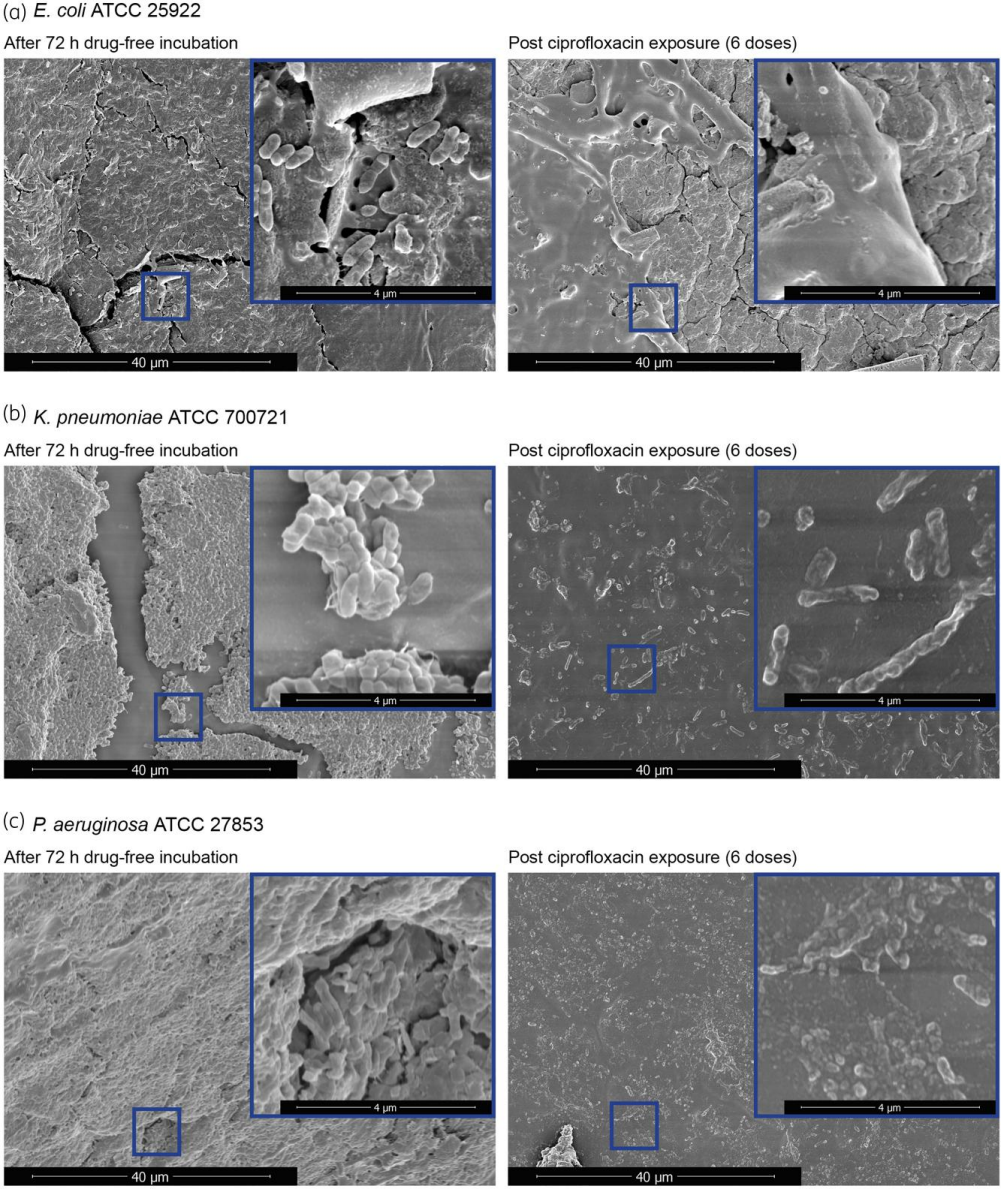
Scanning electron microscopy biofilm images of urinary catheters following incubation with ATCC isolates in the CA-UTI model before and after ciprofloxacin exposure. This figure appears in colour in the online version of *JAC* and in black and white in the print version of *JAC*.

ECO-25922 and PAE-27853 biofilms were completely disrupted, and planktonic bacterial density reduced to below the limit of detection, after the initial 24 h of ciprofloxacin exposure, which was then maintained through the 3 days of therapy. At follow-up, 24 h after cessation of ciprofloxacin, both ECO-25922 and PAE-27853 began to reform biofilms (to 3.3 ± 0.7 and 5.2 ± 0.3 log_10_ cfu/cm^2^, respectively) and regrew in the bladder (to 5.5 and 5.7 log_10_ cfu/mL, respectively) (Figure 2). In contrast, for KPN-700721 the biofilm and planktonic growth was detectable throughout (EOT biofilm mass 3.0 ± 0.6 log_10_ cfu/cm^2^; EOT bacterial density 4.7 log_10_ cfu/mL). After 24 h following cessation of ciprofloxacin, there was robust re-formation of the biofilm and planktonic populations (to 6.7 ± 0.3 log_10_ cfu/cm^2^ and 8.1 log_10_ cfu/mL, respectively) (Figure 2). Biofilm formation and ciprofloxacin-induced disruption were visualized by SEM images after 72 h drug-free incubation and after 3 days of ciprofloxacin exposure (Figure 3).

**Figure 4.**
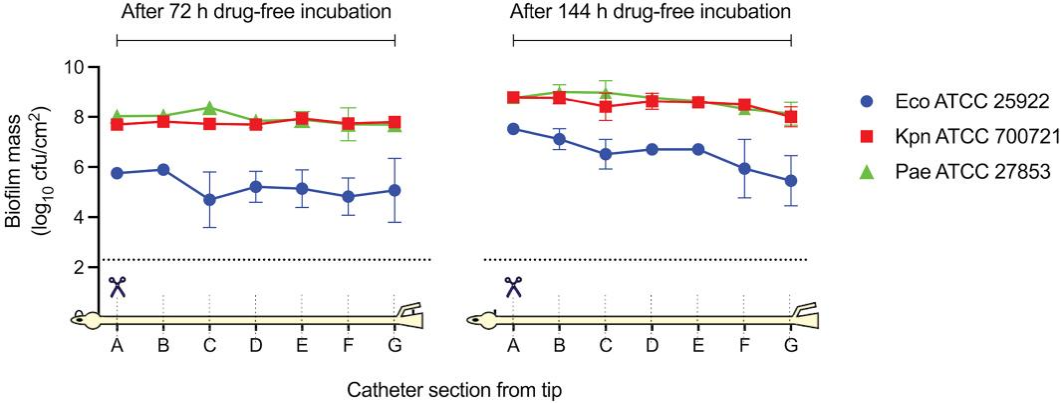
Biofilm coverage along the length of the catheter. Biofilm mass quantification every 5 cm along the entire length of the urinary catheter following drug-free incubation in the CA-UTI model with ATCC strains. Two separate experiments performed with incubation times of 72 and 144 h, respectively. Error bars represent the standard deviation of the mean biofilm mass quantification tested in triplicate. Dotted line represents the limit of detection of the quantitative cultures. Eco, *Escherichia coli*; Kpn, *Klebsiella pneumoniae*; Pae, *Pseudomonas aeruginosa*. This figure appears in colour in the online version of *JAC* and in black and white in the print version of *JAC*.

Extended drug-free incubation demonstrated that the biofilm formation covered the entire length of the urinary catheter, from the catheter tip (section A) to 35 cm (section G) (Figure 4), increasing in mass from 72 to 144 h incubation. Along the length of the catheter, KPN-700721 and PAE-27853 had minimum change in biofilm mass. In contrast, ECO-25922 had a reduction in the biofilm mass from 7.5 ± 0.2 log_10_ cfu/cm^2^ at the tip, to 5.5 ± 1.0 log_10_ cfu/cm^2^ at 35 cm, with increasing non-confluence of biofilm coverage.

### Expanded testing including clinical isolates in the CA-UTI model

#### E. coli

Average biofilm mass was 6.3 ± 1.1 log_10_ cfu/cm^2^ and planktonic bacterial density was 8.1 ± 0.2 log_10_ cfu/mL after 72 h drug-free incubation. No isolate had an RSP identified. One isolate (ECO-132) had reduced biofilm formation (3.5 ± 0.7 log_10_ cfu/cm^2^) despite unrestricted planktonic growth (Table 1).

**Figure 5.**
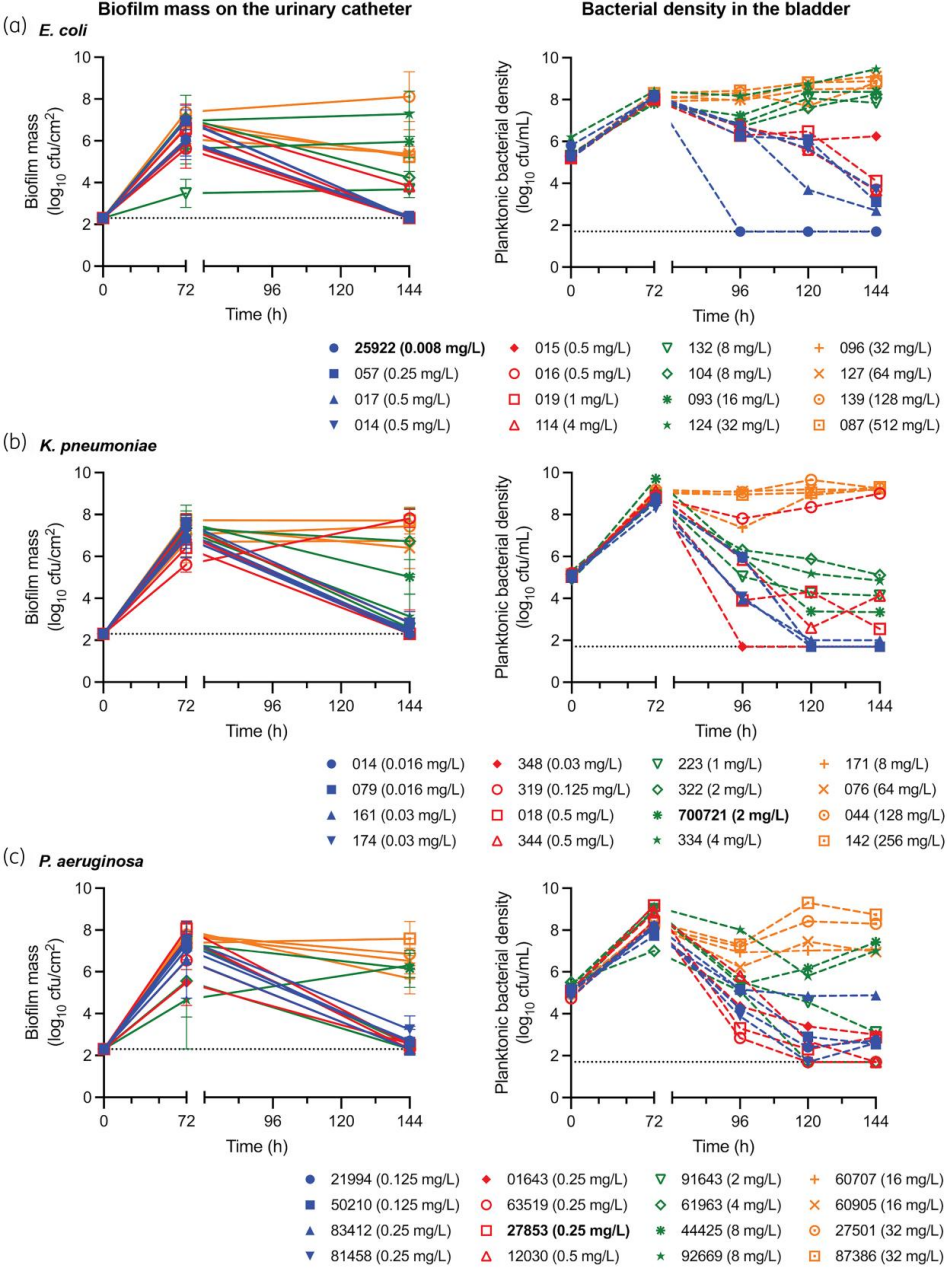
Drug-free incubation and post-exposure to ciprofloxacin in the catheter-associated urinary tract infection model. Biofilm mass (lefthand graphs, solid lines; error bars represent the standard deviation of the mean biofilm mass quantification tested in triplicate) and planktonic bacterial density in the bladder (righthand graphs, dashed lines) for (a) ECO, (b) KPN and (c) PAE clinical isolates and ATCC strains following 72 h drug-free incubation (0–72 h) and 72 h exposure to ciprofloxacin administration equivalent to 750 mg orally 12-hourly for 3 days (72–144 h). The dotted line represents the limit of detection of the quantitative cultures. ATCC strains highlighted in bold. This figure appears in colour in the online version of *JAC* and in black and white in the print version of *JAC*.

Following ciprofloxacin exposure, all susceptible isolates (ciprofloxacin MIC ≤ 0.5 mg/L, *n *= 6) had complete biofilm disruption (no growth in all three segments). However, the activity against the planktonic bacteria was less pronounced: only one isolate had no growth (ECO-ATCC-25922), four isolates had low-level residual growth (ECO-057, ECO-017, ECO-014, ECO-016; range 2.7–3.7 log_10_ cfu/mL), and one isolate had less than 2 log_10_ kill (ECO-015). Of isolates with LLR (ciprofloxacin MIC 1–4 mg/L, *n *= 2), one isolate had complete biofilm disruption (ECO-019), and the other had >3 log_10_ reduction in the biofilm mass (ECO-114). Both isolates had >3 log_10_ kill in the planktonic bacteria. Isolates with HLR (ciprofloxacin MIC >4 mg/L, *n *= 8) all failed to have a significant biofilm mass reduction and failed to kill the planktonic bacteria (Table 2 and Figure 5a).

**Table 2.** Post-exposure endpoint assessment stratified by baseline ciprofloxacin MIC

	*E. coli*			*K. pneumoniae*			*P. aeruginosa*		
		R			R			R	
	S/I	LLR	HLR	S/I	LLR	HLR	I	LLR	HLR
	(MIC ≤ 0.5)	(MIC 1–4)	(MIC > 4)	(MIC ≤ 0.5)	(MIC 1–4)	(MIC > 4)	(MIC ≤ 0.5)	(MIC 1–4)	(MIC > 4)
Biofilm disruption									
No growth^a^	6	1	—	3	—	—	3	1^e^	—
>3 log_10_ kill	—	1	—	4	2	—	4	1	—
1–3 log_10_ kill	—	—	3^c^	—	1	—	1^d^	—	3^f^
<1 log_10_ kill, stasis, no kill	—	—	5	1^d^	1	4	—	—	3
Planktonic bacterial kill									
No growth^b^	1	—	—	4	—	—	2	1^e^	—
>3 log_10_ kill	4	2	—	3	4	—	6	1	—
1–3 log_10_ kill	1	—	—	—	—	—	—	—	4^g^
<1 log_10_ kill, stasis, no kill	—	—	8	1^d^	—	4	—	—	2

I, susceptibile increased exposure; R, resistant; S, susceptible standard dosing regimen.

^a^No growth in all three catheter segments, limit of detection 2.3 log_10_ cfu/cm^2^.

^b^Limit of detection 1.7 log_10_ cfu/mL.

^c^ECO isolates with HLR that had some degree of biofilm kill (1–3 log_10_ reduction) included ECO-104 (MIC 8), ECO-096 (MIC 32) and ECO-087 (MIC 512).

^d^KPN*-*319 (MIC 0.125 mg/L) was an outlier due to the presence of a ciprofloxacin RSP at baseline (RSP MIC 16 mg/L).

^d^PAE-01643 appeared to have had less biofilm kill at EOT; however, in the setting of drug-free growth restriction (biofilm mass 5.5 log_10_ cfu/cm^2^) the average EOT biofilm mass of 2.9 log_10_ cfu/cm^2^ (one segment no growth, other segments 2.6 and 2.8 log_10_ cfu/cm^2^) greater limits the capacity to achieve >3 log_10_ kill, despite the EOT result reflecting a >50% change in biofilm mass.

^e^PAE-61963 had complete biofilm disruption and kill in the planktonic population at EOT; however, this isolate was shown to have drug-free growth restriction (biofilm mass 5.6 log_10_ cfu/cm^2^; bacterial density 7.0 log_10_ cfu/mL).

^f^PAE isolates with HLR that had some degree of biofilm kill (1–3 log_10_ reduction) included PAE-44425 (MIC 8), PAE-60707 (MIC 16) and PAE-60905 (MIC 16).

^g^PAE isolates with HLR that had some degree of planktonic kill (1–3 log_10_ reduction) included PAE-44425 (MIC 8), PAE-92669 (MIC 8), PAE-60707 (MIC 16) and PAE-60905 (MIC 16).

Emergence of ciprofloxacin resistance (>2-fold log_2_ dilution rise in MIC) was identified in three isolates (ECO-114, ECO-132, ECO-104). ECO-144 and ECO-132 displayed resistance in both the biofilm and planktonic regrowth, and ECO-104 had resistance in the planktonic regrowth only (Table 1).

#### K. pneumoniae

Average biofilm mass formation was 7.1 ± 0.7 log_10_ cfu/cm^2^ and planktonic bacterial density was 8.9 ± 0.3 log_10_ cfu/mL after 72 h drug-free incubation. Baseline RSP was identified in KPN-319 by growth on CIP-MHA-2 mg/L (ciprofloxacin MIC 16 mg/L; RSP:TP ratio of 2.0 × 10^−2^ and 2.5 × 10^−3^ in the biofilm and planktonic populations, respectively). KPN-319 had reduced biofilm formation (5.6 ± 0.4 log_10_ cfu/cm^2^) despite unrestricted planktonic growth (Table 1).

Following ciprofloxacin exposure, of the susceptible isolates (ciprofloxacin MIC ≤ 0.5 mg/L, *n *= 8), three had complete biofilm disruption (KPN-014, KPN-018, KPN-344) and four had residual biofilm growth with >3 log_10_ reduction (KPN-079, KPN-161, KPN-174, KPN-348). KPN-319 was an expected outlier due to the amplification of the RSP. Of isolates with LLR (ciprofloxacin MIC 1–4 mg/L, *n *= 4), two had >3 log_10_ reduction in biofilm mass (KPN-223, KPN-334) and two failed to significantly reduce the biofilm (KPN-ATCC-700721, KPN-322). All had residual growth in the planktonic bacteria (density of 3.3 to 5.1 log_10_ cfu/mL) albeit reflective of a >3 log_10_ kill. Isolates with HLR (ciprofloxacin MIC > 4 mg/L, *n *= 4) all failed to have significant biofilm mass reduction and planktonic kill (Table 2 and Figure 5b).

Emergence of ciprofloxacin resistance was identified in six isolates (KPN-319, KPN-344, KPN-223, KPN-322, KPN*-*ATCC-700721, KPN-171). Half displayed resistance in both biofilm and planktonic regrowth, and half had resistance detected in the RSP of the planktonic regrowth only (Table 1).

#### P. aeruginosa

Average biofilm mass was 7.0 ± 1.2 log_10_ cfu/cm^2^ and planktonic bacterial density was 8.4 ± 0.6 log_10_ cfu/mL after 72 h drug-free incubation. No isolate had an RSP identified. Three isolates formed smaller biofilms with greater non-confluence of biofilm coverage (PAE-92669, 4.7 ± 2.3 log_10_ cfu/cm^2^; PAE-61963, 5.6 ± 1.7 log_10_ cfu/cm^2^; PAE-01643, 5.5 ± 1.1 log_10_ cfu/cm^2^). PAE-61963 also had restricted planktonic growth compared with other isolates (7.0 log_10_ cfu/mL) (Table 1).

Following ciprofloxacin exposure, of the susceptible isolates (ciprofloxacin MIC ≤ 0.5 mg/L, *n *= 8) three had complete biofilm disruption (PAE-83412, PAE-63519, PAE-12030) and four had >3 log_10_ reduction in biofilm mass (PAE-21994, PAE-50210, PAE-81458, PAE-ATCC-27853). The remaining isolate (PAE-01643) had minimal residual biofilm growth at EOT (2.9 log_10_ cfu/cm^2^), although reflective of a >50% change in mass due to the restricted drug-free biofilm growth (5.5 log_10_ cfu/cm^2^). Although all susceptible isolates demonstrated significant kill of the planktonic bacteria, only two isolates had no growth at EOT (PAE-63519, PAE-12030) and the remainder had residual growth ranging from 2.5 to 4.9 log_10_ cfu/mL. Of the isolates with LLR (ciprofloxacin MIC 1–4 mg/L, *n *= 2), one isolate had complete biofilm disruption and planktonic bacterial kill (PAE-61963), and the other had >3 log_10_ biofilm disruption and >3 log_10_ kill in the planktonic population (PAE-91643). Isolates with HLR (ciprofloxacin MIC >4 mg/L, *n *= 6) all failed to have significant biofilm mass reduction and maintained their planktonic bacterial density ≥7.0 log_10_ cfu/mL (Table 2 and Figure 5c).

Emergence of ciprofloxacin resistance was identified in 10 isolates (PAE-50210, PAE-81458, PAE-ATCC-27853, PAE-91643, PAE-44425, PAE-92669, PAE-60707, PAE-60905, PAE-27501, PAE-87386). Isolates with the highest baseline MICs (PAE-60905, PAE-27501, PAE-87386) displayed resistance in both the biofilm and planktonic regrowth. PAE-44425 had resistance in the biofilm RSP regrowth only. The remaining six isolates has resistance in the planktonic regrowth only (Table 1).

### Exposure–response analysis

**Figure 6.**
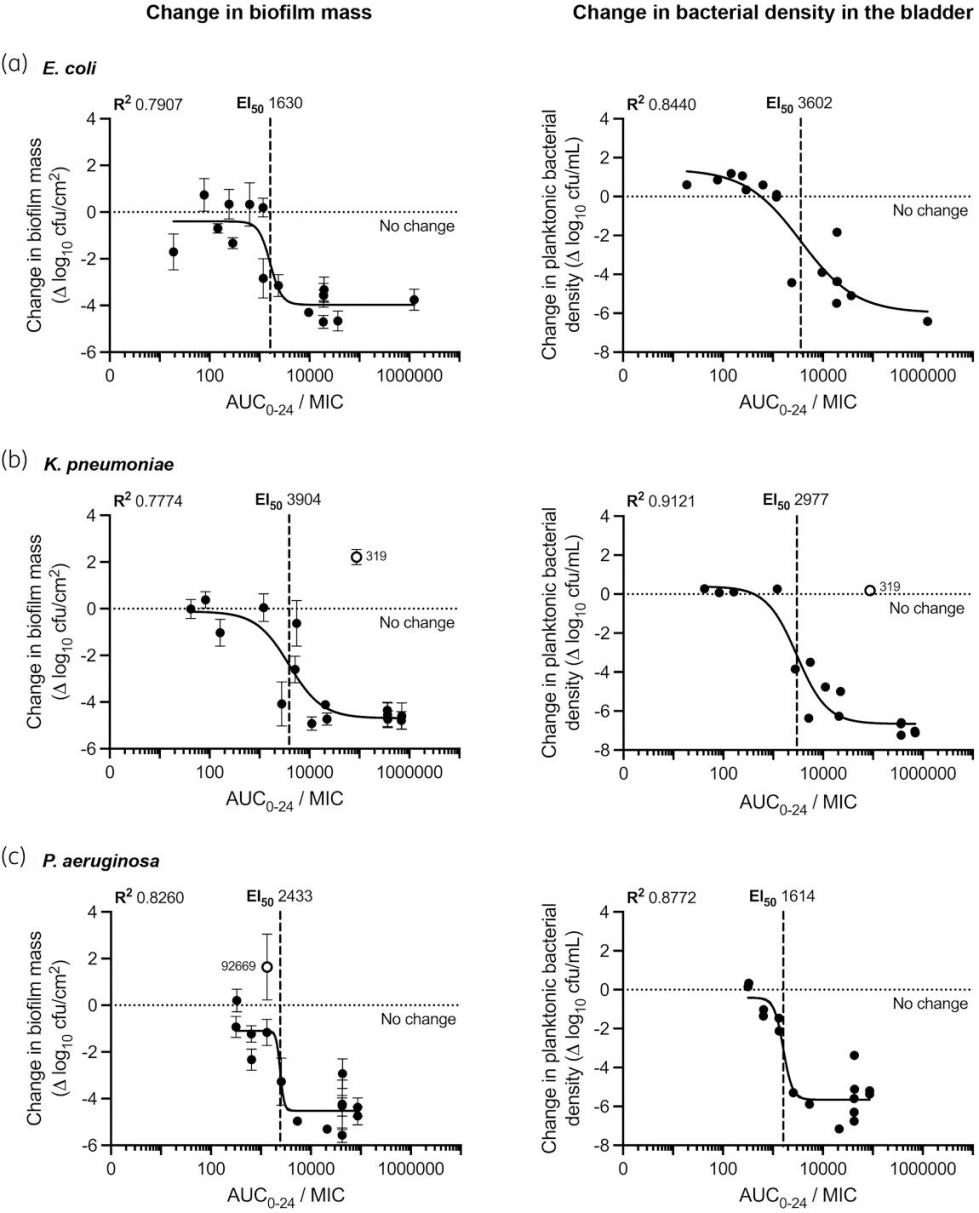
End-of-treatment exposure–response relationships for the change in biofilm mass on the catheter and change in the planktonic bacterial density in the bladder. Non-linear regression variable slope *E*_max_ curves with goodness of fit (*R*^2^) and EI_50_ (vertical dashed line) annotated. Relationship of ciprofloxacin exposure (AUC_0–24_/MIC) and the end-of-treatment PD endpoints for (a) ECO, (b) KPN and (c) PAE isolates. PD endpoints assessed are the change in biofilm mass (left; error bars represent the standard deviation of the mean biofilm mass quantification tested in triplicate) and the change in bacterial density in the bladder (right) compared with the growth after 72 h drug-free incubation. Baseline MIC measurements by standard methodology using CAMHB were used. Excluded isolates from the analyses are identified as open circles and isolate number annotated: KPN-319 due to the presence of an RSP in the baseline population, and PAE-92669 due to reduced biofilm growth after 72 h drug-free incubation and increase at EOT. Horizonal dotted line represents no change in the biofilm mass and bacterial density.

For biofilm disruption, KPN required greater ciprofloxacin exposure (AUC_0–24_/MIC) for a 3 log_10_ reduction in biofilm mass (5858; *R*^2^ = 0.7774) compared with ECO (1630; *R*^2^ = 0.7907) and PAE (2433; *R*^2^ = 0.8260) (Table 3, Figure 6). For planktonic bacterial kill, ECO isolates required greater ciprofloxacin exposure (AUC_0–24_/MIC) for a 3 log_10_ kill in the bladder (5920; *R*^2^ = 0.8440) compared with KPN (2825; *R*^2^ = 0.9121) and PAE (1605; *R*^2^ = 0.8772) (Table 3, Figure 6). Both ECO and PAE had more isolates with low-level persistent growth in the bladder at EOT. Goodness-of-fit assessments were worse using MIC measurements in mSHU (Table S3). Similar PK/PD relationships were found using the EOT biofilm mass and EOT planktonic bacterial density as endpoints (Figure S3). The MCS supported a 95% PTA of achieving both a 3 log_10_ kill in the biofilm and planktonic bacteria for ECO and KPN isolates with MIC ≤ 0.5 mg/L and PAE isolates with MIC ≤ 1 mg/L (Table S4).

**Table 3.** Ciprofloxacin urinary AUC_0–24_/MIC targets for end-of-treatment bacterial response endpoints

	Species	*R* ^2^	3 log_10_ kill	95% CI
Change in biofilm mass at end of treatment	*E. coli*	0.7907	2117	1275–4347
	*K. pneumoniae*	0.7774^a^	5858	1719–18 674
	*P. aeruginosa*	0.8260^b^	2485	1323–4592
Change in planktonic bacterial density at end of treatment	*E. coli*	0.8440	5920	2362–14 544
	*K. pneumoniae*	0.9121^a^	2825	1321–4585
	*P. aeruginosa*	0.8772	1614	1223–2565

A non-linear regression variable slope *E*_max_ model was used. *R*^2^ describes the goodness of fit. No constraints applied. The exposure (AUC_0–24_/MIC) required for a 3 log_10_ kill of the biofilm and planktonic bacterial populations, compared with the growth after 72 h drug-free incubation, is presented with the 95% CI. Baseline MIC measurements by standard methodology using CAMHB were used.

^a^KPN-319 excluded due to the presence of an RSP in the baseline population.

^b^PAE-92669 excluded due to reduced biofilm growth after 72 h drug-free incubation and increase at end of treatment.

## Discussion

We demonstrated biofilm formation and ciprofloxacin-induced disruption in three uropathogen species in a novel CA-UTI model. Following 3 days of ciprofloxacin treatment, we found that ECO planktonic growth frequently persisted in the bladder, KPN biofilms were comparatively more difficult to disrupt, and PAE had a greater propensity for emergence of ciprofloxacin resistance. Our results highlight the challenges antimicrobial therapy alone has for treating CA-UTIs and provides an *in vitro* model able to quantify exposure–response relationships.

In the literature, plasma ciprofloxacin *f*AUC/MIC targets for 2 log_10_ kill are 140 in a neutropenic thigh model.^19^ Less is known about urine-specific targets. Ciprofloxacin is predominantly renally excreted and achieves high urinary concentrations. However, high interindividual variation in fluid intake, urodynamics, urinary chemical make-up and urinary ciprofloxacin concentrations highlight the importance of considering urine-specific PK/PD to ensure dosing recommendations will encompass all individuals. The impact of pH on the efficacy of antimicrobials is particularly relevant for UTI PK/PD, where the acidification of urine may be recommended for the prevention and treatment of UTIs. Ciprofloxacin antibacterial activity is significantly reduced in urine of low pH and high MgSO_4_.^20–23^ The reduced uptake of fluoroquinolones into bacterial cells has been reported as the most probable mechanism for the observed increase in MIC measurements and neutralization of activity in time–kill experiments.^24^ Similarly, impairment of *in vitro* activity has been reported for other antimicrobials despite uropathogen growth remaining unaffected, most notably for *E. coli*.^25–27^ In contrast, a murine UTI model showed no correlation between urine pH and the efficacy of ciprofloxacin and fosfomycin.^28^ Importantly, not all antimicrobials are impaired by low pH, with nitrofurantoin demonstrating an increase in *in vitro* bactericidal activity at lower pH levels,^29^ and fosfomycin PK/PD simulations showing improved target attainment with a decrease of pH from 7.0 to 6.0 associated with changes in physicochemical properties.^30^

Ciprofloxacin activity against ECO in a uUTI model required AUC_0–24_/MIC targets of 1521 and 2383 for a 3 log_10_ kill at EOT and 1 log_10_ kill at follow-up (24 h after ceasing ciprofloxacin), respectively.^17^ The experimental differences in the uUTI simulation are the urodynamics (bladder volume increases over time with intermittent voiding), the starting inoculum prior to ciprofloxacin administration (6 versus 8 log_10_ cfu/mL), and the altered urinary concentration–time curve due to bladder volume changes. These data supported the use of high-dose ciprofloxacin against urinary isolates with LLR. In comparison, in the CA-UTI model ciprofloxacin activity against ECO showed the exposure required for a 3 log_10_ kill at EOT in the planktonic bacteria was higher (AUC_0–24_/MIC = 5920). This supports the notion that treatment of a CA-UTI is more challenging than a uUTI. Similar conclusions can be extrapolated to KPN, where biofilm disruption (AUC_0–24_/MIC = 5858) would only be possible for isolates with MIC ≤ 0.5 mg/L with high-dose therapy. For PAE, the AUC_0–24_/MIC targets for both biofilm disruption and planktonic kill were comparatively lower (2485 and 1605, respectively), supporting efficacy for isolates with MIC ≤ 1 mg/L, although our observation of emergence of ciprofloxacin resistance could limit treatment efficacy.

In comparison with murine UTI/pyelonephritis models, studies have demonstrated variable ciprofloxacin bactericidal activity with infections caused by ECO strains with ciprofloxacin LLR, in isolates containing *qnr* and *aac(6′)-Ib-cr* genes and *gyrA*/*gyrB* mutations, when simulating ciprofloxacin dosing corresponding to 500 mg orally 12-hourly for 2 to 3 days (with respect to serum concentrations).^31–34^ In the most recent paper,^31^ four ECO isolates were examined: a WT isolate tested against a range of ciprofloxacin dosing regimens, and three LLR isolates with *gyrA* mutations treated with ciprofloxacin 7 mg/kg (0.2 mg) four times daily per mouse (starting 24 h after bacterial inoculation; terminated 24 h after last dose). PK showed a urinary *C*_max_ of 553 mg/L, but a comparatively lower urinary AUC_0-24_ of 2572 mg·h/L compared with that simulated in the *in vitro* model. Although isolates with LLR had reduction in urinary bacterial counts, complete eradication was not achieved. This same ciprofloxacin dosing was used by the same authors in 2012,^33^ with isogenic ECO strains with/without *qnr* genes. Ciprofloxacin was again shown to be less efficient in reducing bacterial density in urine in the LLR strains, although bacterial kill was still evident with an EOT median bacterial density in urine reduced to ∼2 log_10_ cfu/mL, whereas the WT strain had complete eradication.

These comparisons highlight the challenges in translating *in vitro* and *in vivo* findings to clinical practice. Although *in vitro* models lack host factors, they have the advantage of directly mimicking human PK exposures at the site of infection to directly elucidate bacterial exposure–response relationships. In contrast, animal models require sophisticated scaling in relation to dosing, PK and elimination. *In vitro* models can also examine more variables than would be possible in animals, collect a greater number of samples and run longer experiments. Compared with humans, mice do not naturally acquire UTIs, have limited bladder urine storage, void more frequently, and have a vesicourethral reflux that can lead to kidney infection. Furthermore, differences in bladder anatomy, host response to infection, urine composition, and altered expression of bacterial virulence factors further limit the translation of findings.^35^ Interestingly, a recent CA-UTI mouse model was able to recapitulate the pathophysiology of human infections, demonstrating inflammation due to catheterization and the contribution of fibrinogen to biofilm formation, suggesting this model could have an important role in translational research.^36^

CA-UTIs involve bacteria within biofilms. These are complex microbial communities that have marked heterogeneity and adaptative characteristics, where antimicrobials and host immune activity are impeded and can lead to treatment failures.^37^ Standardized biofilm susceptibility endpoint parameters, such as the minimal biofilm eradication concentration, the minimal biofilm inhibitory concentration, the biofilm bactericidal concentration or the biofilm prevention concentration, are needed to improve the clinical validity of antibiofilm assays.^38^ Further advancements in model design would be the inclusion of cell penetration assays, such as that used in an immunoresponsive 3D urine-tolerant human urothelial model.^39^ Future studies could use this CA-UTI model to examine emerging therapies, including strategies for prevention (e.g. surface modifications, cyclic diguanylate inhibitors) and eradication (e.g. promote dispersal, target persister cells, combination therapy with bacteriophages).^10,40^

The limitations of our study should be considered. Firstly, we examined monomicrobial infections, whereas in clinical practice polymicrobial CA-UTI infections are more common, especially co-occurrence with *Enterococcus faecalis*.^41–43^ Secondly, we did not investigate longer durations of ciprofloxacin therapy. Our treatment was for 3 days, whereas guidelines recommend 7–14 days.^44^ The dosing regimen was selected for practical reasons and to allow comparison with prior testing in an uncomplicated UTI simulation. Thirdly, we started treatment without changing the urinary catheter. However, it has been demonstrated that patients with long-term urinary catheters have multiple bacteria persistently co-colonizing catheterized individuals, despite catheter changes and antibiotic use.^45^ Fourthly, bacteria were only allowed 72 h to form biofilms prior to exposure to ciprofloxacin, which may be insufficient for the formation of fully mature biofilm structures, and biofilm quantification was determined only by conventional cfu enumeration, which may fail to generate reproducibly reliable results.^46^ Fifthly, although we used a synthetic urine medium, it is not possible to completely replicate the entire composition of human urine. Furthermore, the acidity of mSHU (pH 5.6) required for chemical solubility would reduce ciprofloxacin activity and may not reflect the most commonly encountered urinary pH in humans. We also noted different changes in media pH after static drug-free incubation in mSHU compared with pooled human urine. Nonetheless, any changes in pH in the bladder compartment in the CA-UTI model would likely be mitigated by the continuous media inflow and outflow. Finally, our model lacks the host immune response and the urothelial cellular structure into which uropathogens can invade and form intracellular bacterial communities.

In summary, using a novel CA-UTI model, we demonstrated and quantified the key factors implicated in CA-UTI treatment failures, including biofilms resistant to antimicrobial-induced disruption (seen in KPN isolates), bacterial persistence in the bladder (seen in ECO isolates), and the emergence of antimicrobial resistance (seen in PAE isolates). To advance the clinical translation of our findings, our novel CA-UTI model can be further applied to examine other variables, such as polymicrobial CA-UTIs, longer durations of antimicrobial therapy and novel therapeutic strategies.

## Supplementary Material

dkae424_Supplementary_Data

## Supplementary data

Figures S1–S3 and Tables S1–S4 are available as Supplementary data at *JAC* Online.
