# Supplementary material for: Oral ciprofloxacin biofilm activity in a catheter-associated urinary tract infection model
Source: J Antimicrob Chemother. 2024 Nov 29;80(2):413–26. doi: 10.1093/jac/dkae424 (PMC11787899; doi:10.1093/jac/dkae424)
Supplement: dkae424_Supplementary_Data [file dkae424_supplementary_data.docx]

**SUPPLEMENTARY MATERIAL**

**SUPPLEMENTARY METHODS**

**Static growth curves in different media**

Drug-free growth of ATCC strains (*E. coli* 25922, *K. pneumoniae* 700721, *P. aeruginosa* 27853) was performed in CAMHB, pooled human urine, and mSHU. A starting inoculum of 4 log_10_ cfu/mL was prepared in 25 mL of each media and incubated for 24 h at 36°C with vigorous shaking (200 rpm). Growth curves were repeated, and media pH was measured before and after incubation. Due to limited availability, a different batch of pooled human urine (thawed from frozen stock) was used for the repeat static growth experiment for pH measurements.

**CA-UTI model set-up**

Drug distribution equations were used to inform the *in vitro* flow rate, compartment volumes and amount of ciprofloxacin administered to achieve the target urinary PK parameters. The model comprises of a fresh media reservoir, an intestinal compartment, a circulatory compartment, and sixteen bladder compartments, connected via autoclavable tubing with flow rates controlled by four peristaltic pumps. The fresh media reservoirs (10 L every 24 h), containing mSHU, was set at 400 mL/h to flow into the intestinal compartment (900 mL) and then onto the circulatory compartment (1450 mL). The volumes in the intestinal and circulatory compartments were kept static. The media flow was 25 mL/h into each of the sixteen bladder compartments. Each bladder compartment was prepared in 100 mL glassware with a urinary catheter (single-use, latex foley urinary catheter, 14 Ch/Fr [4.7 mm], Bard®, USA) aseptically placed in the outflow position, and the volume maintained at 50 mL. Compartments were held in a thermoregulated water bath at 37°C on a multi-hotplate-stirrer. Ciprofloxacin dosing schedules were administered as a 3-day course, to simulate 750 mg orally 12-hourly, administered directly into the intestinal compartment.

**Pharmacokinetic sampling**

For each CA-UTI experiment, forty *in vitro* PK samples were each collected during ciprofloxacin dosing, from 72 to 144 h, including 9 samples from three representative bladder compartments at the C_max_ and C_min_ of each day of dosing, 2 h before and 2 h after the C_max_ on the third day of dosing, and at the completion of the experiment (at 144 h). Additionally, the remaining 13 bladder compartments were sampled at the C_max_ on the third day of treatment. Average ciprofloxacin exposure (AUC_0-24_) was calculated from the measured concentrations for each experiment. Each isolate’s estimated AUC_0-24_/MIC exposure was proportionally calculated from the measured C_max_ compared to the average C_max_ for that experiment.

PK samples were filtered, stored at -80°C, and batched for testing. Ciprofloxacin concentrations were measured by an ultra-high performance liquid chromatography with fluorometric detection (UHPLC-Fl) method on a Nexera2 liquid chromatograph connected to a RF-20Axs fluorescence detector (Shimadzu, Kyoto, Japan). Calibration range was 0.1 to 1000 mg/L. Ciprofloxacin has previously been shown to be stable when incubated in SHU, with 0.2%, 1.9% and 4.6% reduction in the measured concentration at 24, 48 and 72 h, respectively.^1^ Inter-day and inter-compartment variability was assessed by the average of the relative standard deviation. Linear regression and Bland-Altman plots quantified the accuracy and bias of the measured concentrations compared to theoretical target values.

1. Abbott IJ, van Gorp E, Cottingham H, Macesic N, Wallis SC, Roberts JA, Meletiadis J, Peleg AY. Oral ciprofloxacin activity against ceftriaxone-resistant Escherichia coli in an in vitro bladder infection model. J Antimicrob Chemother. 2023 Feb 1;78(2):397-410. doi: 10.1093/jac/dkac402. PMID: 36473954; PMCID: PMC9890216.

**Scanning electron microscopy (SEM)**

Biofilm formation and CIP-induced disruption was visualised by SEM for the ATCC strains. A segment from the dissected catheter underwent an initial washing step and then was fixed with a 2.5% glutaraldehyde solution, washed with a 0.1M cacodylate buffer, and further fixed with 1% osmium tetraoxide. Segments were washed in MilliQ water and dehydrated with several ethanol (50%, 70%, 90%, 95% and 100%) and hexamethyldisilazane solutions (25%, 50%, 75% and 100%). Segments were copper coated with a sputter coater (SCD005, BAL-TEC) and viewed under a FEI Nova NanoSEM 450. Images were taken at 100x, 1000x and 10000x magnification.

**Quantification of the biofilm mass and the planktonic bacterial density**

During the initial experiments with ATCC strains, biofilm formation was assessed at 6, 24, 48 and 72 h during drug-free incubation, at 96, 120 and 144 h during ciprofloxacin exposure, and at 168 h one day after the completion of ciprofloxacin therapy. The extent of the coverage of the biofilm along the length of the catheter was determined after 72 and 144 h drug-free incubation. For the subsequent experiments including clinical strains, biofilm mass was assessed at two time-points: following drug-free incubation at 72 h, and at the end of treatment after 3-days of ciprofloxacin administration at 144 h. ATCC strains underwent repeat testing with the clinical strains.

Prior to processing, urinary catheters were removed from the bladder outflow and a section cut above the catheter balloon at approximately 2 cm from the drainage end of the catheter. This catheter section was divided into 4 equal segments by dissecting horizontally and vertically. For the initial experiments with the ATCC strains, the section was 1 cm in length (each quartered segment curved internal surface measurement was 0.5 x 0.3 cm). For the subsequent experiments, the section size was increased to 1.6 cm to improve accuracy of the dissection procedure (each quartered segment curved internal surface measurement was 0.8 x 0.3 cm). For the ATCC strains, an additional six segments were collected every 5 cm along the entire length of the catheter after 72 h and 144 h drug-free incubation.

To quantify the biofilm mass (cfu/cm^2^), 3 of the 4 segments were washed by repeated submersion in sterile PBS (Sigma-Aldrich) to remove non-adherent bacteria and placed into an Eppendorf tube containing 1 ml of PBS. To release the biofilm adhered on the catheter surface, segments were vortexed for 30 seconds at maximum settings (2700 RPM) three times followed by sonication at 42kHz for 10 minutes, before a final vortex for 30 seconds. Quantitative cultures were performed by serial 10-fold dilutions of each processed sample, with 20 µL plated from each dilution plated onto MHA (drug-free MHA, CIP-MHA-2 mg/L, and CIP-MHA-128 mg/L plates) and incubated aerobically, 35 ±1°C for 16-20 h. Ciprofloxacin supplemented plates were re-incubated for an additional 24 h.

At each dilution, growth of a single colony, pin-point colonies, or haze were not considered true growth and disregarded. The LOD for biofilm mass quantification was 2.5 log_10_ cfu/cm^2^ for the initial experiments with the shorter (0.5 x 0.3 cm) catheter segments, and 2.3 log_10_ cfu/cm^2^ for the subsequent experiments with longer (0.8 x 0.3 cm) catheter segments. The average biofilm mass was determined from the three segments with the mean (± SD) reported. Where the standard deviation of the mean biofilm mass was greater than ±1 log_10_ cfu/cm^2^, non-confluent biofilm growth was recorded.

The planktonic bacterial density in the bladder was assessed by sampling directly from the media in each bladder compartment. This was performed at 0 and 72 h during drug-free incubation, and at 96, 120 and 144 h during ciprofloxacin exposure. For the initial experiments with the ATCC strains, an additional time-point after the completion of ciprofloxacin therapy was collected at 168 h. Prior to processing, antibiotic carry-over was negated by a centrifuge/wash process that was performed twice. The serial dilution and plating procedure followed the same procedure as described for the biofilm mass quantification, detailed above. The limit of detection (LOD) was considered as 1.7 log_10_ cfu/mL.

**SUPPLEMENTARY TABLES**

**Table S1. Chemical components of modified synthetic human urine (mSHU).**

| **Chemical** | | **g/L** |
| --- | --- | --- |
| Sodium chloride | NaCl | 5.844 |
| Sodium sulphate | Na_2_SO_4_ | 2.4147 |
| Urea | Urea | 16.8168 |
| Potassium chloride | KCl | 2.8329 |
| Calcium chloride | CaCl_2_ | 0.4439 |
| Creatinine | Creatinine | 1.0181 |
| Citric acid trisodium salt dihydrate | Na_3_C_6_H_5_O_7_ | 1.9999 |
| Ammonium chloride | NH_4_Cl | 1.0698 |
| Magnesium sulphate | MgSO_4_ | 0.3852 |
| Sodium oxalate | Na_2_C_2_O_4_ | 0.0241 |
| Sodium phosphate monobasic | NaH_2_PO_4_ | 0.5616 |
| Sodium phosphate dibasic | Na_2_HPO_4_ | 0.9227 |
| Potassium dihydrogen phosphate | KH_2_PO_4_ | 2.1774 |
| Uric acid | C_5_H_4_N_4_O_3_ | 0.1009 |
| Sodium bicarbonate | NaHCO_3_ | 1.1341 |
| Magnesium chloride hexahydrate | MgCl_2_·6H_2_O | 0.6506 |
| Lactic acid | C_3_H_6_O_3_ | 0.0991 |
| Ferrous sulphate heptahydrate | FeSO_4_·7H_2_O | 0.0014 |
| 20% (w/v) casamino acids ^a^ | - | 0.1 % (v/v) |
| 10% (w/v) yeast extract ^b^ | - | 0.1% (v/v) |

^a^, Bacto^TM^ Casamino Acids (Thermo Fisher Scientific, #223050). ^b^, Bacto^TM^ Yeast Extract (Thermo Fisher Scientific, #212750). Media adjusted to pH 5.6 and filter sterilised.

**Table S2. Impact of media on ciprofloxacin MIC baseline testing**

| **Isolate #** | **Ciprofloxacin MIC (mg/L)** | | | |
| --- | --- | --- | --- | --- |
|  | **CAMHB** | | **mSHU** | |
|  | **Median MIC** | **Range** | **Median MIC** | **Range** |
| ***E. coli*** | | | | |
| 25922^a^ | 0.008 | (0.008) | 0.125 | (0.125) |
| 057 | 0.25 | (0.25) | 8 | (8) |
| 017 | 0.5 | (0.25-0.5) | 8 | (8) |
| 014 | 0.5 | (0.25-0.5) | 8 | (8) |
| 015 | 0.5 | (0.5) | 8 | (8) |
| 016 | 0.5 | (0.5) | 8 | (8) |
| 019 | 1 | (1) | 16 | (16) |
| 114 | 4 | (4-8) | 256 | (256) |
| 132 | 8 | (4-8) | 64 | (64) |
| 104 | 8 | (8) | 256 | (256) |
| 093 | 16 | (16) | 512 | (512) |
| 124 | 32 | (32) | 512 | (512) |
| 096 | 32 | (32) | 512 | (512) |
| 127 | 64 | (64) | >512 | (>512) |
| 139 | 128 | (128) | 512 | (512) |
| 087 | 512 | (512) | >512 | (>512) |
| ***K. pneumoniae*** | | | | |
| 014 | 0.016 | (0.016 - 0.03) | 0.5 | (0.5) |
| 079 | 0.016 | (0.016) | 0.5 | (0.5) |
| 161 | 0.03 | (0.03) | 0.5 | (0.5 - 1) |
| 174 | 0.03 | (0.016 - 0.06) | 0.5 | (0.5) |
| 348 | 0.03 | (0.03) | 0.5 | (0.5) |
| 319^b^ | 0.125 | (0.06 - 0.125) | 8 | (8) |
| 018 | 0.5 | (0.5) | 16 | (8 - 16) |
| 344 | 0.5 | (0.5) | 8 | (8) |
| 223 | 1 | (0.5 - 1) | 8 | (8) |
| 322 | 2 | (2) | 32 | (32) |
| 700721^a^ | 2 | (1 - 2) | 32 | (32 - 64) |
| 334 | 4 | (4) | 128 | (128) |
| 171 | 8 | (8) | 128 | (128) |
| 076 | 64 | (32 - 64) | >512 | (>512) |
| 044 | 128 | (128) | >512 | (>512) |
| 142 | 256 | (256) | >512 | (>512) |
| ***P. aeruginosa*** | | | | |
| 21994 | 0.125 | (0.125 - 0.25) | 2 | (2 - 4) |
| 50210 | 0.125 | (0.125 - 0.25) | 1 | (1 - 2) |
| 83412 | 0.25 | (0.125 - 0.25) | 2 | (2 - 4) |
| 81458 | 0.25 | (0.125 - 0.5) | 4 | (2 - 4) |
| 01643 | 0.25 | (0.125 - 0.5) | 4 | (2 - 4) |
| 63519 | 0.25 | (0.25) | 4 | (4) |
| 27853^a^ | 0.25 | (0.25 - 0.5) | 4 | (4) |
| 12030 | 0.5 | (0.5) | 8 | (8 - 16) |
| 91643 | 2 | (2-4) | 128 | (128) |
| 61963 | 4 | (4) | 64 | (64) |
| 44425 | 8 | (4 - 8) | 128 | (128 - 256) |
| 92669 | 8 | (8 - 16) | 512 | (512) |
| 60707 | 16 | (16) | 512 | (512) |
| 60905 | 16 | (16) | 512 | (512) |
| 27501 | 32 | (32) | 512 | (512) |
| 87386 | 32 | (32 - 64) | 512 | (512) |

MIC testing in triplicate by broth microdilution, median MIC and range presented. ^a^, ATCC strains. MIC measurements are higher when tested in mSHU, with an average bias of 4.1 ± 1.0 two-fold dilutions higher compared with testing in CAMHB.

**Table S3. End of treatment bacterial response PK/PD targets using MIC measurement in mSHU**

|  | **Species** | **R^2^** | **3 log_10_ kill** | **95% CI** |
| --- | --- | --- | --- | --- |
| **Change in biofilm mass at end of treatment** | *E. coli* | 0.6573 | 300 | 145 – *n/a* |
|  | *K. pneumoniae* | 0.7259^a^ | 402 | 84 – 1915 |
|  | *P. aeruginosa* | 0.6674^b^ | 89 | 25 – 791 |
| **Change in planktonic bacterial density at end of treatment** | *E. coli* | 0.7498 | 331 | 61 – 2055 |
|  | *K. pneumoniae* | 0.8680^a^ | 135 | 45 – 297 |
|  | *P. aeruginosa* | 0.8543 | ~82^c^ | *n/a* – *n/a* |

EOT, end of treatment. Non-linear regression variable slope E_max_ model was used. R^2^ describes the goodness of fit. No constraints applied. The exposure (AUC_0-24_/MIC_mSHU_) required for a 3 log_10_ kill of the biofilm and planktonic bacterial populations, compared with the growth after 72 h drug-free incubation, is presented with the 95% confidence interval (CI). Baseline MIC measurements by broth microdilution using modified synthetic human urine (mSHU). ^a^, KPN-319 excluded due to the presence of a RSP in the baseline population. ^b^, PAE-92669 due to reduced biofilm growth after 72 h drug-free incubation and increase at EOT. ^c^, non-linear regression curve had an ambiguous fit. *n/a*, the upper or lower confidence interval for the interpolated value was not possible.

**Table S4. PTA of achieving 3 log_10_ biofilm disruption and planktonic kill**

MCS applying AUC_0-24_/MIC targets for PD endpoints of biofilm disruption (3 log_10_ reduction in biofilm mass) and planktonic kill (3 log_10_ reduction in bacterial density in bladder) at EOT. Expected urinary exposure following oral ciprofloxacin 750mg 12-hourly with ±50% allowance to account for variability in human urinary concentrations. CIP, ciprofloxacin.

**SUPPLEMENTARY FIGURES**

**Figure S1. Bacterial growth kinetics in different media**


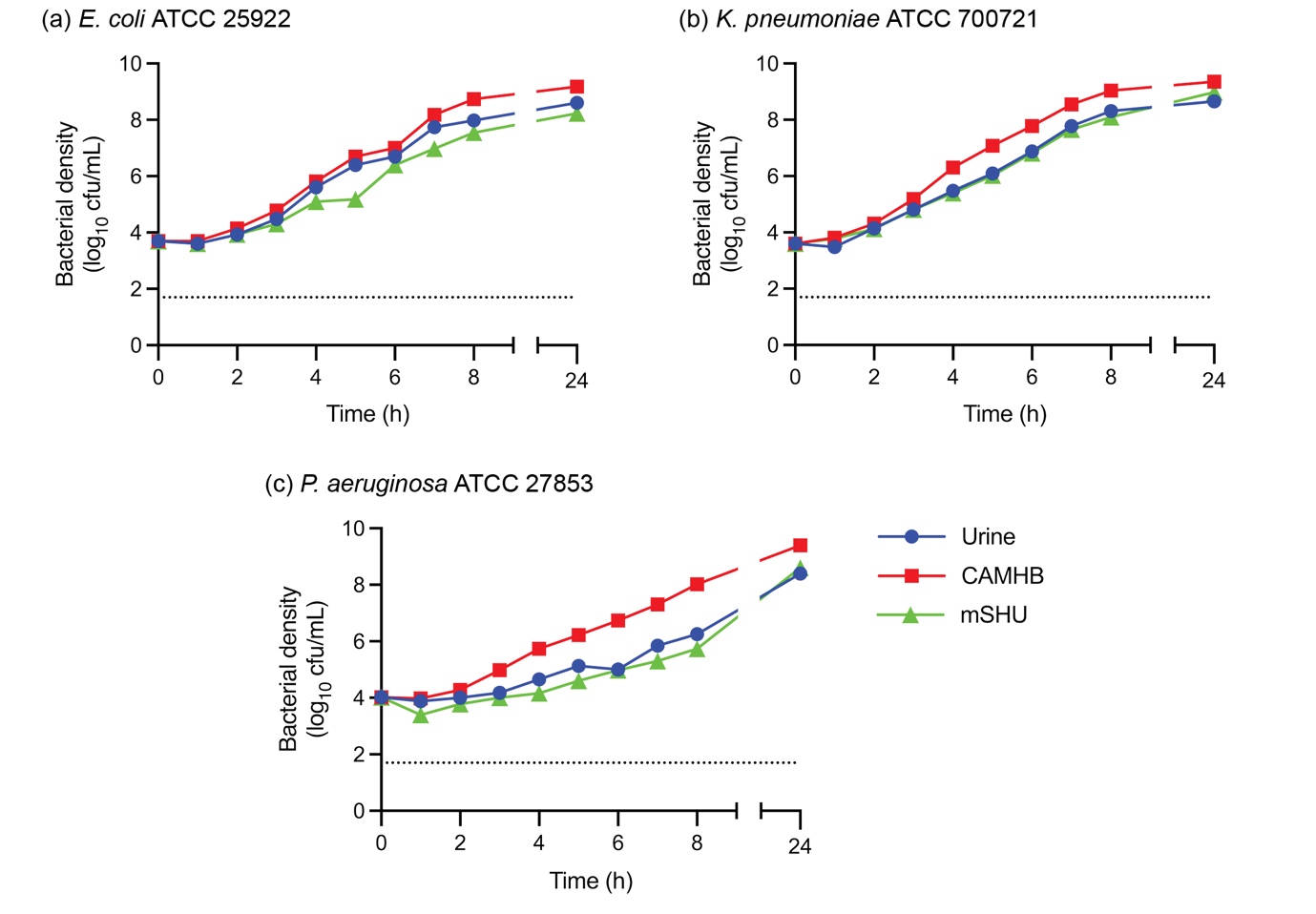


Drug-free growth of ATCC strains in pooled human urine (blue circles), cation-adjusted Mueller-Hinton II broth (CAMHB, red squares), and modified synthetic human urine (mSHU, green triangles) assessed after 24 h static incubation (36°C with vigorous shaking, 200 rpm) in 25 mL of each media. The dotted line represents the limit of detection of the quantitative cultures. Prior to incubation media pH were CAMHB pH 7.4, urine pH 7.0, mSHU pH 5.6. After incubation, pH measurements were, for *E. coli*: CAMHB pH 7.1, urine pH 7.2, mSHU pH 5.6; for *K. pneumoniae*: CAMHB pH 7.0, in urine pH 6.9, in mSHU pH 8.5; for *P. aeruginosa*: CAMHB pH 8.1, urine pH 8.1, mSHU pH 6.4.

**Figure S2. *In vitro* ciprofloxacin concentration measurements**


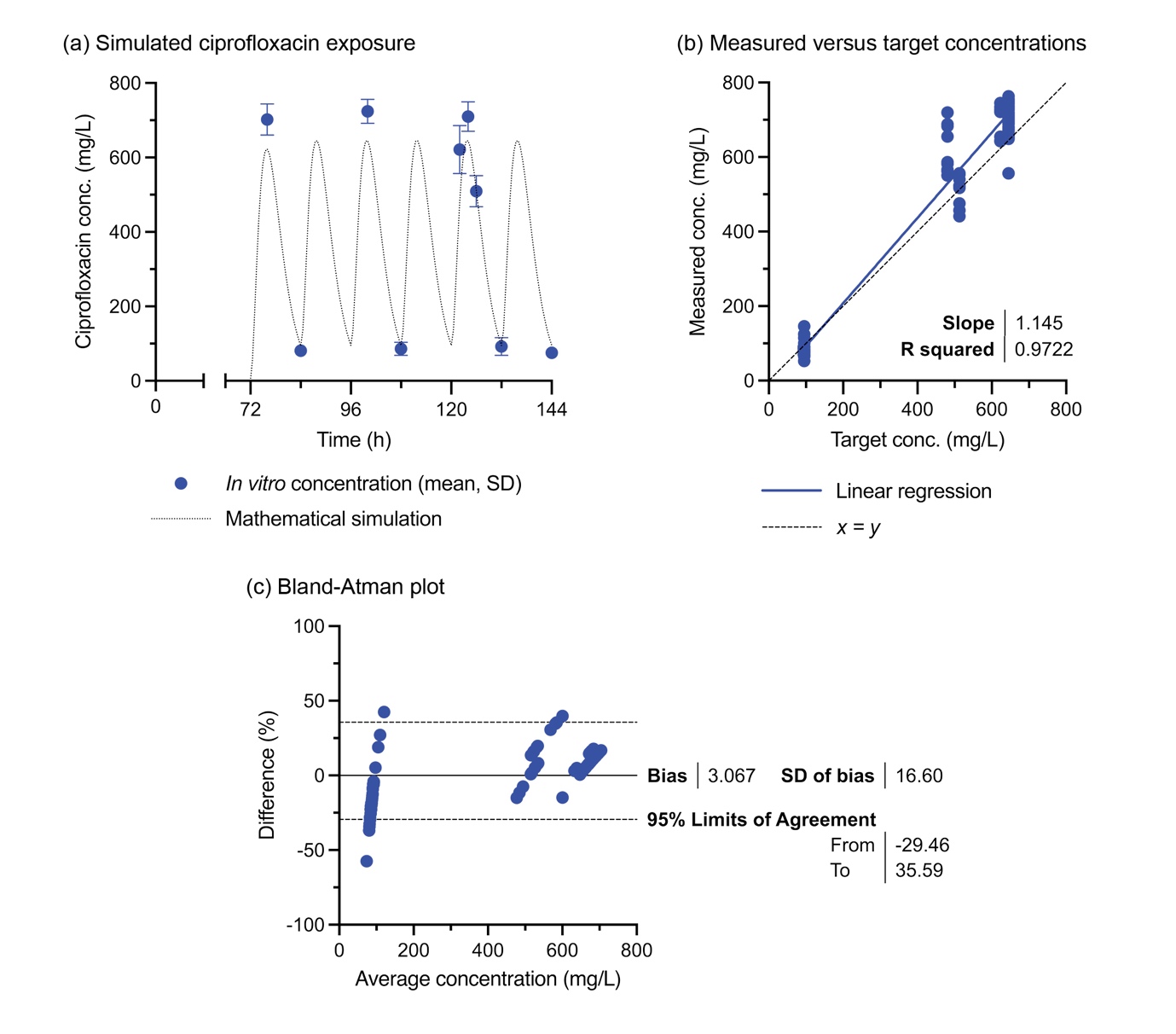


Bland-Altman plot percentage bias of the measure concentration compared with the target is determined by the calculation: 100 × (measured – target)/average) vs. average.

**Figure S3. Exposure-response relationships for biofilm mass on the catheter and the bacterial density in the bladder at the end of treatment**


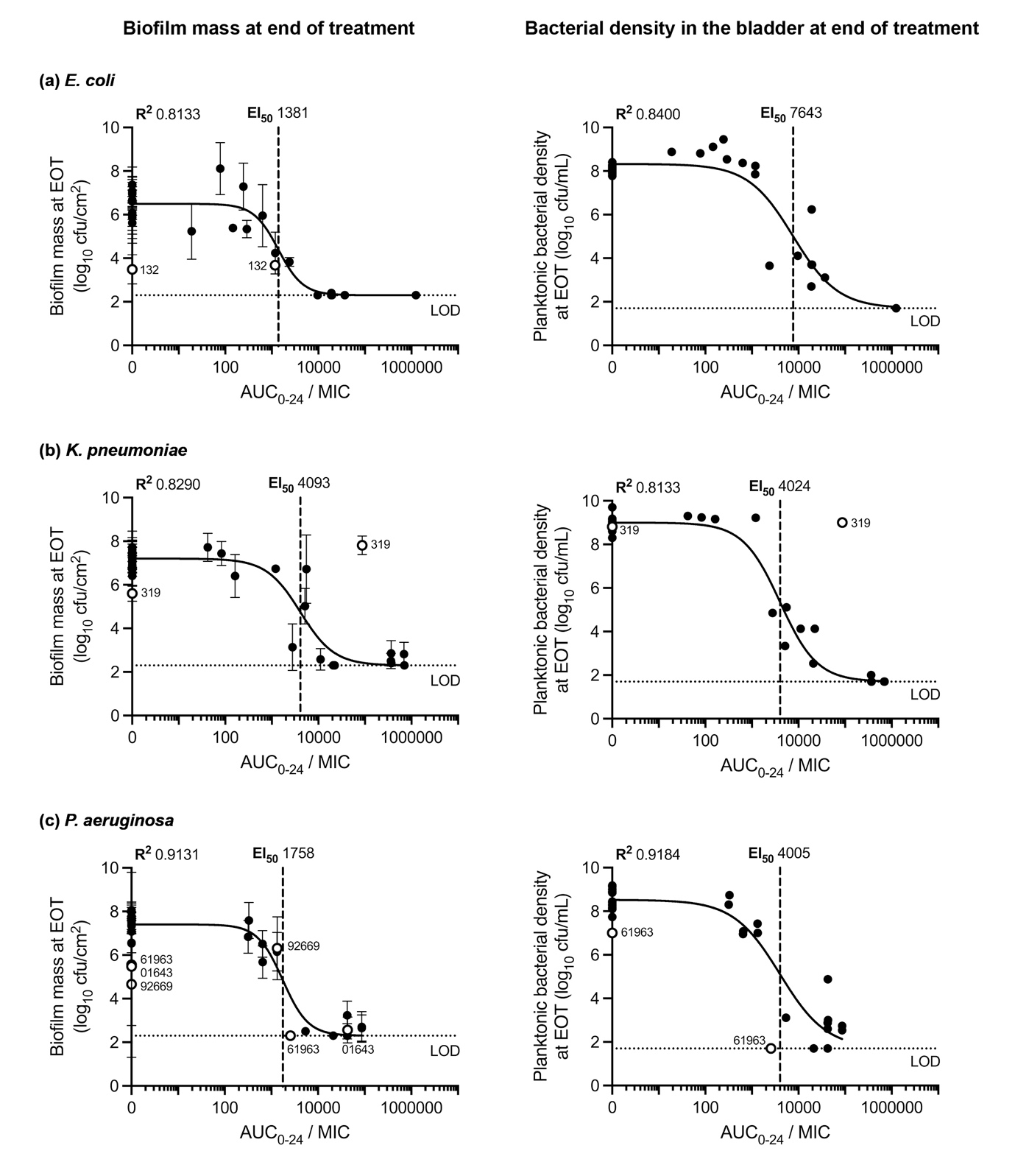


Non-linear regression variable slope E_max_ curves with goodness of fit (R^2^) and EI_50_ (vertical dashed line) annotated. Quantification of the relationship of ciprofloxacin exposure (AUC_0-24_/MIC) and the end of treatment bacterial endpoints for (a) ECO, (b) KPN, and (c) PAE isolates. Endpoints assessed were the quantified biofilm mass (left) and the quantified bacterial density in the bladder (right) at 144 h. Curves constrained bottom to limit of detection (LOD). X=0 data points represent the growth capacity after 72 h drug-free incubation. Baseline MIC measurements by standard methodology using CAMHB were used. Isolates excluded from each analysis are identified as open circles and isolate number annotated: ECO-132 biofilm growth excluded due to reduced drug-free growth; KPN-319 biofilm and planktonic growth excluded due to the presence of a RSP in the baseline population; PAE-01643, PAE-61963, and PAE 92669 biofilm growth excluded due to reduced drug-free growth; PAE-61963 planktonic growth excluded due to reduced drug-free growth. Horizonal dotted line represents the LOD of the quantitative culture of the biofilm mass (left) and the bacterial density (right).
